# Syphilis Communicated by a Kiss

**Published:** 1866-06

**Authors:** 


SYPHILIS COMMUNICATED BY A KISS.—At a recent meeting of the Chicago Medical Society a member related the history of a young woman, whose irreproachable character left no doubt of the truth of her narrative, who experienced the horrors of syphilitic inoculation, through a kiss from the gentleman to whom she was engaged. A chancre upon the lip was the result of this caress, and subsequent medical investigation revealed the fact that the young man was at that time under treatment for syphilitic ulceration of the throat.

